# Ferroptosis participates in neuron damage in experimental cerebral malaria and is partially induced by activated CD8^+^ T cells

**DOI:** 10.1186/s13041-022-00942-7

**Published:** 2022-06-20

**Authors:** Jiao Liang, Yan Shen, Yi Wang, Yuxiao Huang, Jun Wang, Qinghao Zhu, Guodong Tong, Kangjie Yu, Wei Cao, Qi Wang, Yinghui Li, Ya Zhao

**Affiliations:** 1 Department of Medical Microbiology and Parasitology, Fourth Military Medical University, 169# Changle West Road, Xi’an, 710032 China; 2 College of Life Sciences, Northwest University, Xi’an, China; 3 Department of Pathology, Air Force Hospital of Eastern Theater, Nanjing, China; 4 Second Student Brigade, School of Basic Medical Sciences, Fourth Military Medical University, Xi’an, China

**Keywords:** ACSL4, CD8^+^ T cell, Cerebral malaria, Ferroptosis, GPX4, Neuron, TfR1

## Abstract

Cerebral malaria is the most serious complication of malaria infection, with 26% of surviving children having neurological sequelae, which may be caused by neuron damage, but the mechanism is not clear. Ferroptosis has been reported to play an important role in neuron damage in several nervous system diseases. However, the occurrence of ferroptosis in experimental cerebral malaria (ECM) pathogenesis is still unknown. In this study, we firstly detected increased levels of malondialdehyde (MDA) and iron, which are indicators of ferroptosis, in the cerebrum of ECM mice. Some important regulators of ferroptosis, including upregulated expression of transferrin receptor 1 (TfR1) and acyl-CoA synthetase long-chain family member 4 (ACSL4), and downregulation of glutathione peroxidase 4 (GPX4) levels, were also confirmed in ECM mice. Consistently, neuron damage, which was detected in the cerebrum of ECM mice, was positively correlated with reduced GPX4 expression and furtherly rescued by administration of the ferroptosis inhibitor ferrostatin-1 (Fer-1). In addition, primary neurons were damaged by activated CD8^+^ T cells, an effect that was also partially rescued by Fer-1 on amyloid precursor protein expression and mitochondrial membrane potential levels in vitro. Activated CD8^+^ T cells were also shown to infiltrate the cerebrum of ECM mice and upregulate TfR1 expression in primary neurons, which may be an important event for inducing ferroptosis in ECM. Altogether, we show that ferroptosis contributes to neuron damage in ECM pathogenesis, and activated CD8^+^ T cells may be important inducers of neuronal ferroptosis. Hence, targeting ferroptosis may be a promising adjuvant therapeutic strategy for neurological sequelae in patients with cerebral malaria.

## Introduction

Cerebral malaria (CM) is a life-threatening neurological complication of *Plasmodium falciparum* infection, which can lead to fever, seizures, coma, and ultimately death, that has a high incidence rate in children under 5 years of age in sub-Saharan Africa [1]. Although CM can be treated with effective antimalarial drugs, approximately 26% of the surviving children have residual neurological sequelae [2, 3]. Unfortunately, the underlying mechanisms contributing to these neurological sequelae remain poorly understood, which currently hinders any improvement in patient adjuvant therapy.

In general, disruption of the blood–brain barrier, subsequent infiltration of immune cells, and hemorrhage are known to comprise a critical mechanism of CM that can cause nerve tissue damage [4]. Previous research has shown that neuronal damage can lead to neurological sequelae in children with CM and mice with experimental cerebral malaria (ECM) [5–7], but the underlying mechanism remains unclear. Recently, significantly increased levels of oxidative stress were detected in patients with severe malaria, including those with CM [8, 9]. Moreover, administration of antioxidants protects mice with ECM from oxidative stress and neuroinflammation [10, 11]. Therefore, oxidative stress may play a key role in neuronal damage that results in CM neurological sequelae [8, 9, 12], but further studies are warranted to better understand its underlying mechanism. Noteworthy, lipid peroxidation, an important source of oxidative stress, has emerged as an important regulator of cell death through a distinct form of programmed cell death, named ferroptosis, which is closely correlated with several pathological processes, including those of cancer, ischemia–reperfusion injury, and neurodegenerative disorders [13, 14].

Ferroptosis is mediated by iron-dependent lipid peroxidation, triggered by the accumulation of lipid hydroperoxides (L-OOH). Importantly, upregulated expression of transferrin receptor 1 (TfR1), which mediates iron import [15–17], and of acyl-CoA synthetase long-chain family member 4 (ACSL4), which catalyzes the production of L-OOH from polyunsaturated fatty acids [18–20], increase the production of L-OOH, whereas decreased expression of glutathione peroxidase 4 (GPX4), which transforms toxic L-OOH into non-toxic lipid alcohols, has the opposite effect [21, 22]. Ultimately, excessive L-OOH, which is a form of lipid reactive oxygen species, causes rapid and unrepairable damage to membranes and leads to cell death. However, whether ferroptosis participates in the pathogenesis of CM remains unknown.

In this study, we explored the role of ferroptosis in the pathogenesis of ECM and the effect of ferroptosis inhibitors (ferrostatin-1) to protect against neuronal damage, which might be a potential therapeutic strategy to prevent the occurrence of neurological sequelae in patients with CM.

## Materials and methods

### Induction of experimental cerebral malaria

Four-week-old male C57BL/6 mice were purchased from the animal center of Fourth Military Medical University and housed under specific pathogen-free (SPF) conditions with a 12 h light/dark cycle. *Plasmodium berghei* ANKA strain (PbA) was maintained in our laboratory. All mice were randomly assigned into different groups, and experimental mice were infected intraperitoneally (i.p.) with 1 × 10^6^ pRBCs (parasitized-RBCs) to induce Experimental Cerebral Malaria (ECM), as previously reported by our group [23]. For ECM + Fer-1 group, mice were intraperitoneally injected with 5 mg/kg ferrostatin-1 (Fer-1, HY-100579, MCE, USA) on the 1st day, 3rd day, 5th day after *Plasmodium* infection, and the mice in ECM and control group were received an equal volume of PBS. On the 7th day, mice were sacrificed, and the brain tissues were collected for further data analysis.

### Isolation and culture of primary cortical neuron

Brains were immediately collected from newborn mice within 24 h of birth and then dissected. The cerebral cortex was isolated and the meninges and surface vessels were removed. Cortical tissues were rinsed in cold Hank’s buffered saline solution and cut into small pieces, digested with 0.25% trypsin–EDTA for 10 min at 37 °C. The triple volume of Neurobasal A medium (10888-022, Gibco, USA) with 10% fetal bovine serum (FBS, 16140063, Gibco, USA) was added, and the tissue pieces were mechanically separated into single cells by passing through a Pasteur pipette. Cells were pelleted and resuspended in Neurobasal A medium containing 2 mM Glutamine (25030-164, Gibco, USA), 2% B-27 supplement (17504-044, Gibco, USA), and 1% penicillin and streptomycin (14140-148, Gibco, USA), and then plated on Poly-l-lysine (25988-63-0, Sigma, USA)-coated plates. Cultures were incubated in a 5% CO_2_ incubator at 37 °C. Sixty percent of the culture medium was replaced with fresh medium every 3 days. Approximately 2 weeks later, primary neurons were treated with naive-CD8^+^ T or activated-CD8^+^ T cells for 24 h and the morphological changes were observed under the Microscope.

### Purification of spleen CD8^+^ T cells

Spleens were collected from control or ECM mice, and then rinsed in cold HBSS, grind to single cells through a 70 μm-screen. Cells were pelleted and resuspended in Erythrocyte lysate (AR1118, Bosterbio, USA), and then pelleted again to collect white blood cells. CD8^+^ T cells were purified from white blood cells through the magnetic bead sorting kit (558471, BD, USA) according to the manufacturer’s instructions.

### Assessment of lipid peroxidation

Protein samples from mouse brain tissue were extracted by using the RIPA lysis buffer (AR0102, Bosterbio, USA) containing 1% cocktail (4693116001, Roche, USA). The protein concentration of each sample was measured by BCA Protein Assay Kit (P0012, Beyotime, China). The level of malondialdehyde (MDA) was detected through MDA Assay Kit (S0131S, Beyotime, China), according to the manufacturer’s instructions.

### Assessment of Fe contents

Protein samples for Fe content assay were extracted by using Glass homogenizer with 0.9% NaCl solution. The protein concentration was measured by BCA Protein Assay Kit. The Fe content of each sample was detected through Tissue Fe Assay Kit (A039-2-1, Nanjing Jiancheng Bioengineering Institute, China).

### Western blot analysis

Protein samples were extracted using PIPA lysis buffer as above, and then each equal amount protein sample was separated by 10% SDS-PAGE gel and transferred onto PVDF membrane. Then the membranes were blocked with 5% skim milk for 1 h at room temperature, followed by incubation with the primary antibody against TfR1 (ET1702-06, HUABIO, China), ACSL4 (sc-271800, Santa Cruz, USA), GPX4 (ET1706-45, HUABIO, China), APP (25524-1-AP, Proteintech, China), MAP2 (17490-1-AP, Proteintech, China), and β-actin (66009-1-Ig, Proteintech, China) overnight at 4 °C, and then the secondary antibody DyLight 680-labbed Goat Anti-Mouse IgG and DyLight 800-labbed Goat Anti-Rabbit IgG (A23910, A23920, KPL, USA) for 1 h at room temperature avoiding light, and scanned at 700 nm and 800 nm using Odyssey Clx Image. The relative protein expression of TfR1, ACSL4, and GPX4 was calculated using gray value, that was analyzed through Quantity one software and then standardized to β-actin.

### Quantitative real-time polymerase chain reaction (RT-PCR) assay

RNA was isolated from the mouse brain (cerebrum and brainstem) via standard Trizol extraction followed by isopropanol precipitation. The cDNA was synthesized using Reverse Transcription SuperMix (R222-01, Vazyme, China), and then RT-PCR was performed with 2 × SYBR qPCR Master Mix (Q311-02, Vazyme, China) according to the manufacturer’s instruction. The following primers were used: TfR1 Forward Primer 5′-GTTTCTGCCAGCCCCTTATTAT-3′ and Reverse Primer 5′-GCAAGGAAAGGATATGCAGCA-3′; ACSL4 Forward Primer 5′-TGTGCATCCCGC-GATGATT-3′ and Reverse Primer 5′-AGTCCAGGGATACGTTCACAC-3′; GPX4 Forward Primer 5′-TGTGCATCCCGCGATGATT-3' and Reverse Primer 5′-CCCTGTACTTATCCAGGCAGA-3′; GAPDH Forward Primer 5′-TGTGCATCCCGCGATGATT-3′ and Reverse Primer 5′-CCCTGTAC-TTATCCAGGCAGA-3′. The relative mRNA expression of TfR1, ACSL4, and GPX4 was calculated via the 2^−ΔΔCT^ method and standardized to GAPDH.

### Mitochondrial membrane potential (MMP) assay

Primary neurons were seeded into a 96-well plate and cultured for approximately 2 weeks. The neurons were treated with activated-CD8^+^ T cells for 24 h, and with or without Fer-1 (5 μM) pretreatment for 2 h. Then, the MMP probe JC-1 (HY-15534, MCE, USA) was added into the culture with the final concentration of 2 μM and incubated at 37 °C for 30 min. After that, supernatants were discarded and cells were washed twice with PBS, and finally, the Red and Green Fluorescence were measured using Microplate Reader with excitation/emission wavelengths of 458/615 nm and 458/538 nm respectively. The relative MMP was calculated via Red Fluorescence/Green Fluorescence.

### Immunohistochemical staining

Whole brains of mice were removed without meningeal after perfusion and fixed in 4% paraformaldehyde (PFA) overnight. Then the paraffin-embedded sections (5 μm-thick) were prepared after dehydration and transparency, and dehydration was carried out with xylene and downgrade alcohol series. Antigen retrieval was performed in citrate buffer using a heat-induced retrieval method. Subsequently, the tissue sections were incubated with 3% H_2_O_2_ and blocked with blocking buffer (containing 3% BSA, 2% bovine serum, and 0.2% Triton X-100) for 30 min at room temperature, then incubated with the primary antibody (Synaptophysin: GB11553, Servicebio, China; GPX4: ET1706-45, HUABIO, China) overnight at 4 °C, and with Goat Anti-Rabbit IgG secondary antibody (G1213, Servicebio, China) for 1 h at room temperature. Sections were stained with 3,3′-Diaminobenzidine (DAB, A690009-0025, BBI, CAN), counterstained with hematoxylin (E607317-0100, BBI, CAN), and examined using digital slide scanning (Pannoramic DESK, P-MIDI, P25), analyzed using CaseViewer 2.4 (3DHISTECH).

### Immunofluorescence staining

The paraffin sections of mice brains were prepared as above. After antigen retrieval, the tissue sections were blocked with blocking buffer, then incubated with the primary antibody Anti-GPX4 (ET1706-45, HUABIO, China) and Anti-NeuN (66836-1-Ig, Proteintech, China), or Anti-CD8 (GB13429, Servicebio, China) and Anti-CD3 (60181-1-Ig, Proteintech, China) overnight at 4 °C, and with the secondary antibody for 1 h at room temperature, including Cy3 conjugated Goat Anti-Rabbit IgG (GB21303, Servicebio, China) and FITC conjugated Goat Anti-Moue IgG (GB22301, Servicebio, China). The nuclei were stained with DAPI staining solution (G1021, Servicebio, China) for 10 min. Finally, the slides were mounted in Antifade (G1401, Servicebio, China) and examined using digital slide scanning (Pannoramic DESK, P-MIDI, P25), and analyzed using CaseViewer 2.4 (3DHISTECH). The relative expression of GPX4 and NeuN was calculated using fluorescent density, analyzed through ImageJ software, and then standardized to DAPI count.

### Statistical analysis

All data were analyzed for statistical significance with the unpaired Student’s *t*-test through GraphPad Prism 8.0 program (GraphPad Software Inc., USA) and expressed as means ± standard deviation (SD). Significant differences between groups were analyzed using a one-way analysis of variance, and *p* < 0.05 was considered statistically significant.

## Results

### Ferroptosis may participate in ECM pathogenesis

**Fig. 1:**
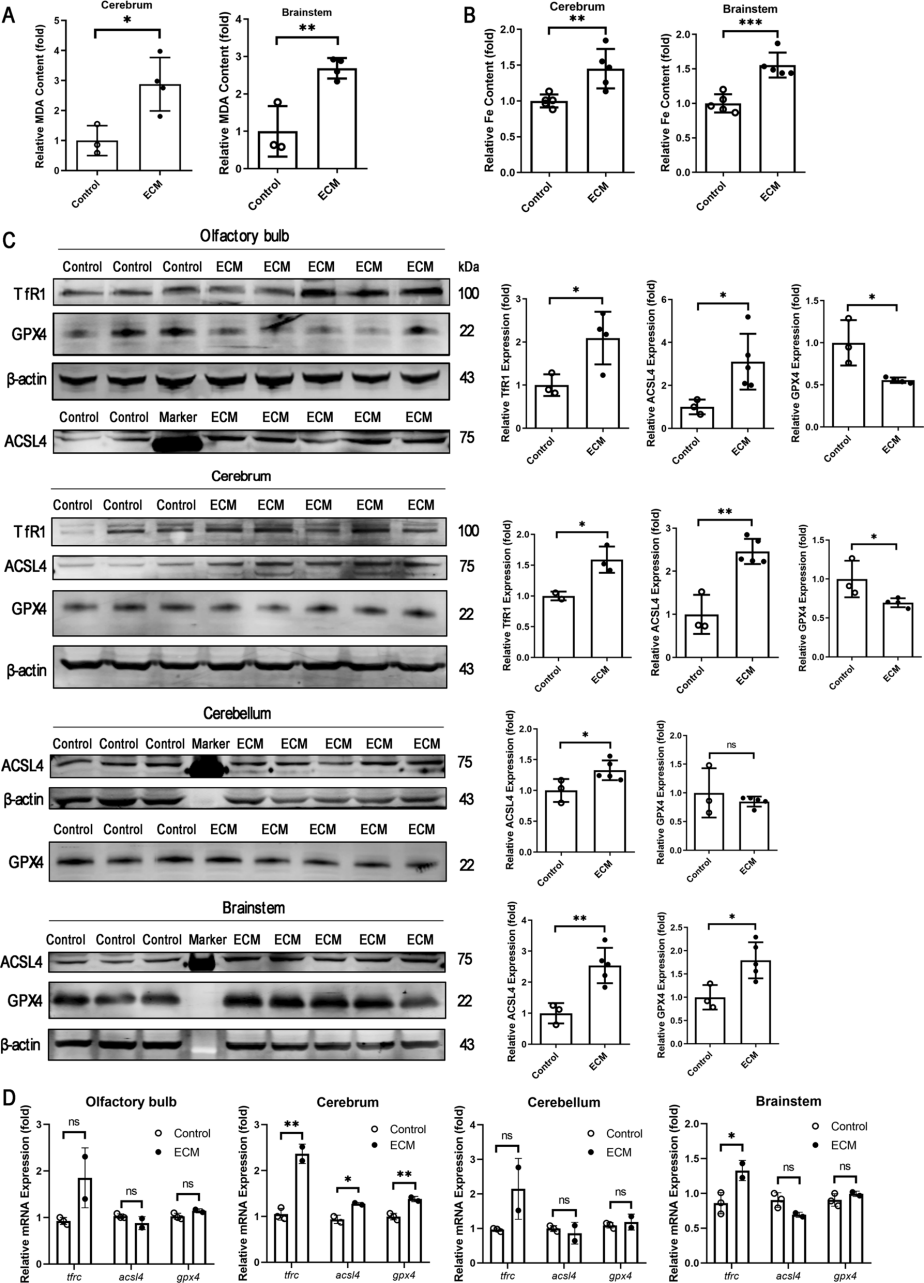
Distinguishing indicators and regulators of ferroptosis in different brain regions of ECM mice. **a** and **b** MDA levels (**a**) and Fe contents (**b**) in the cerebrum and brainstem of control and ECM mice were measured using a commercial kit. **c** Expression of TfR1, ACLS4, and GPX4 in the olfactory bulb, cerebrum, cerebellum, and brainstem of control and ECM mice was measured by western blotting. Relative quantitative analysis was performed densitometry. **d** mRNA expression of *tfrc*, *acsl4*, and *gpx4* in the olfactory bulb, cerebrum, cerebellum, and brainstem of control and ECM mice was measured by quantitative real-time polymerase chain reaction. Relative expression is shown as fold-change in relation to control levels. Data are reported as mean ± standard deviation. Each point represents one animal. *p < 0.05, **p < 0.01, and ***p < 0.001 *vs.* control. *ACSL4* acyl-CoA synthetase long-chain family member 4, *ECM* experimental cerebral malaria, *GPX4* glutathione peroxidase 4, *MDA* malondialdehyde, *TfR1* transferrin receptor 1

We generated a mouse model of ECM and tested the key indicators of ferroptosis, such as levels of lipid peroxidation and iron (Fe) content. The lipid peroxidation marker malondialdehyde (MDA) and Fe contents were remarkably increased in ECM specimens, both in the cerebrum (*p* = 0.0228 and 0.0083, respectively) and brainstem (*p* = 0.0057 and 0.0006, respectively) (Fig. 1a and b). We then analyzed the expression of key regulators of ferroptosis—GPX4, TfR1, and ACSL4—in different brain regions. ACSL4 expression was significantly upregulated in all brain regions of ECM mice (olfactory bulb, *p* = 0.0367; cerebrum, *p* = 0.0013; cerebellum *p* = 0.0381; brainstem, *p* = 0.0058), whereas TfR1 expression was upregulated only in the olfactory bulb (*p* = 0.0351) and cerebrum (*p* = 0.0368) (Fig. 1c). In contrast, GPX4 expression was downregulated in the olfactory bulb (*p* = 0.0196) and cerebrum (*p* = 0.0498), but upregulated in the brain stem (*p* = 0.0211), with no obvious change in the cerebellum of ECM mice (*p* = 0.4557). In agreement with these findings, *tfrc* mRNA levels were upregulated in all brain regions of ECM mice (olfactory bulb, *p* = 0.074; cerebrum, *p* = 0.0029; cerebellum, *p* = 0.0849; brainstem, *p* = 0.042) (Fig. 1d), and upregulated *acsl4* was only detected in the cerebrum of ECM mice (*p* = 0.0365). Moreover, *gpx4* expression was also upregulated in the cerebrum of ECM mice (*p* = 0.0083), which contrasted with its protein levels downregulation, probably because of negative feedback regulation in a GPX4-deficient microenvironment. These results indicate that ferroptosis probably occurs in the brain of ECM mice, especially in the cerebrum via reduced GPX4 expression and increased TfR1 and ACSL4 levels. Therefore, we speculated that brain cells may be damaged by ferroptosis during ECM pathogenesis.

### Neuronal damage in ECM is probably due to GPX4 deficiency

**Fig. 2:**
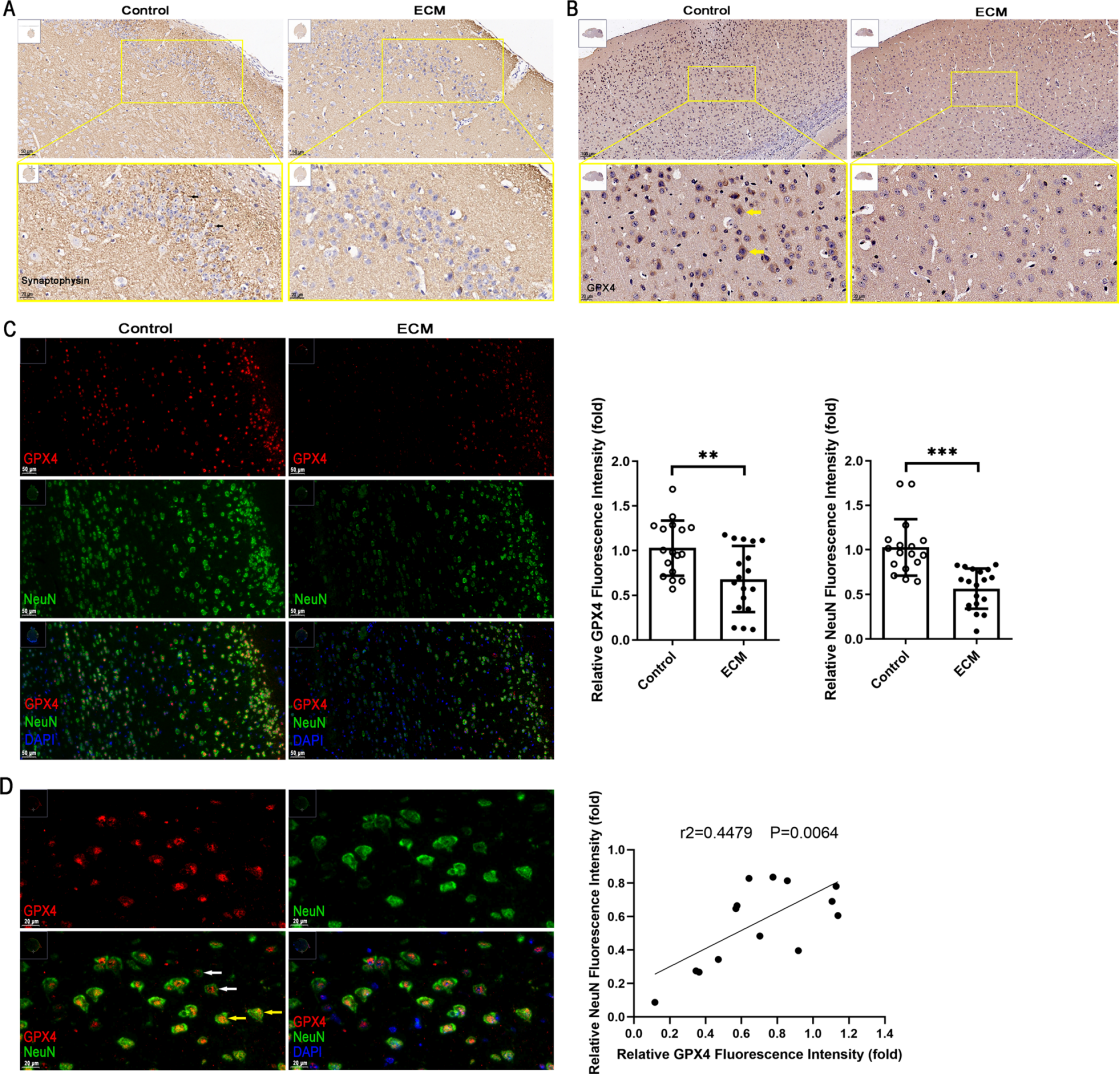
Simultaneous neuronal damage and reduced GPX4 expression in ECM mice. **a** and **b** Expression of synaptophysin (brown, **a**) and GPX4 (brown, **b**) in the cerebrum of control and ECM mice was detected by immunohistochemistry staining. The nucleus was stained with hematoxylin (blue). Black and yellow arrows indicate the high expression of synaptophysin and GPX4, respectively. **c** Expression of GPX4 (red) and NeuN (green) in the cerebrum of control and ECM mice was detected by immunofluorescence staining and quantified by fluorescent density analysis. The nucleus was stained with DAPI (blue). Relative fluorescent density is shown as the fold-change in relation to control levels. Data are reported as the mean ± standard deviation. Each point represents one region of the immunofluorescence staining image. **p < 0.01 and ***p < 0.001 *vs.* control. **d** Correlation between GPX4 and NeuN levels in the cerebrum of ECM mice was analyzed based on the fluorescent density of different regions in immunofluorescence-stained sections. White and yellow arrows indicate low and high levels of GPX4 and NeuN, respectively. Each point represents one staining region of ECM. *ECM* experimental cerebral malaria, *GPX4* glutathione peroxidase 4, *NeuN* neuronal nuclei

Neuronal damage is the main risk factor contributing to neurological sequelae in the pathogenesis of CM. Thus, we evaluated neuron damage in ECM mice by immunohistochemical staining of synaptophysin. The areas with high expression of synaptophysin corresponded to putative synapses in the cerebrum of control mouse brain, whereas synaptophysin expression was significantly reduced in ECM (Fig. 2a), which suggested that the neurons were damaged. Previous research has indicated that GPX4 is the only enzyme that neutralizes L-OOH and plays an essential role in inducing ferroptosis in various diseases. As expected, GPX4 expression in the cerebrum of ECM mice was significantly decreased (Fig. 2b), which was also consistent with the aforementioned results. Next, analysis of the GPX4 expression in neurons by immunofluorescence staining showed diminished neuronal nuclei (NeuN) and GPX4 staining in the cerebrum of ECM mice as compared with that of control mice (Fig. 2c). Fluorescence density quantification in the continuous images confirmed that NeuN and GPX4 levels were significantly decreased in ECM cerebrum (*p* < 0.0001 and *p* = 0.0049, respectively), suggesting that the neurons were seriously damaged in these mice, which may be related to GPX4 downregulation. Furthermore, we found that GPX4 expression was markedly reduced in the damaged neurons of ECM mice (low expression of NeuN) (Fig. 2d). Correlation analysis of the fluorescence density in continuous images revealed a positive correlation between NeuN and GPX4 expression (*p* = 0.0064). Hence, these results indicate that neuronal damage is probably caused by GPX4 deficiency in ECM mice, which may lead to L-OOH accumulation and induce ferroptosis.

### Inhibition of ferroptosis ameliorates lipid peroxidation and neuronal damage in ECM

**Fig. 3:**
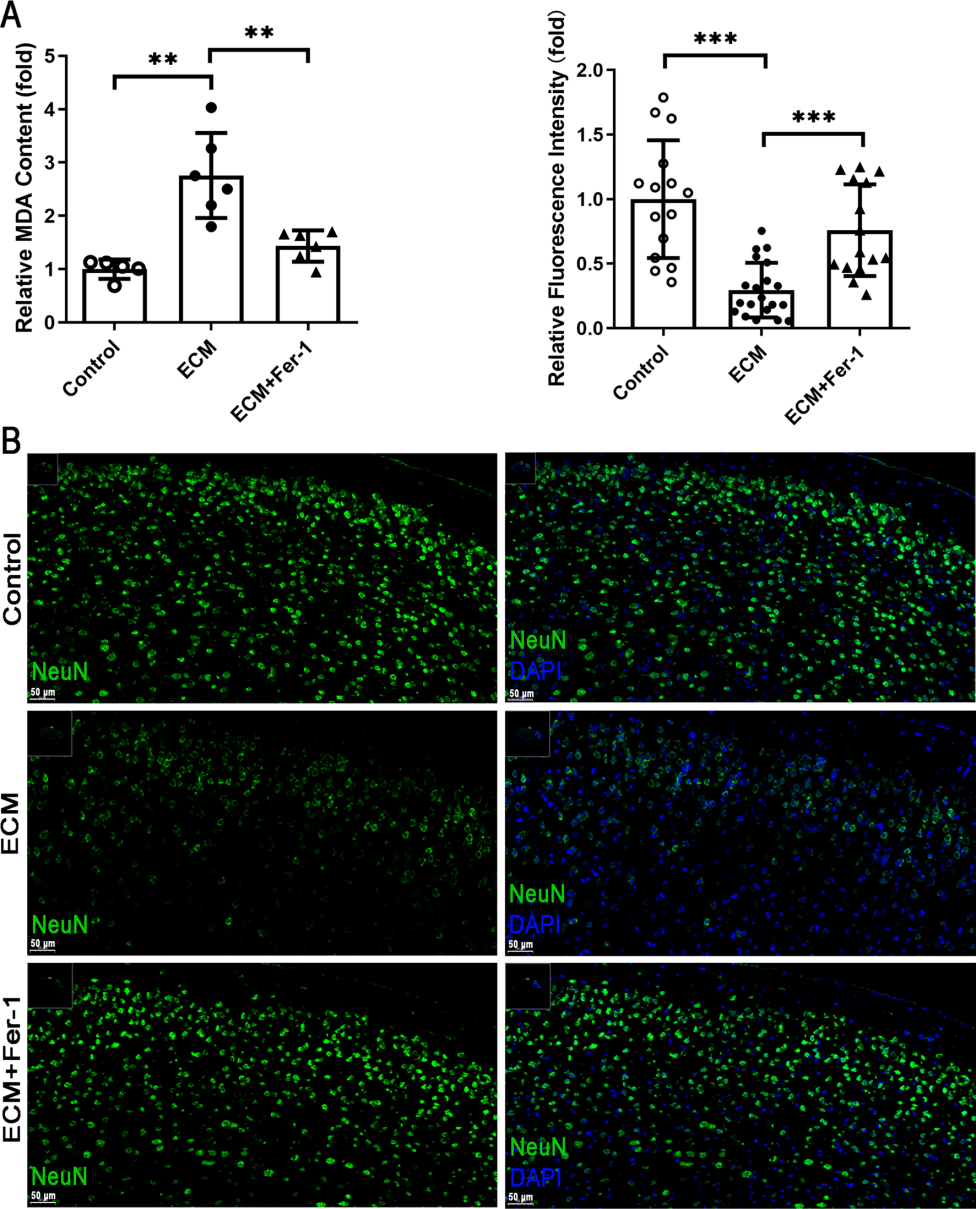
Fer-1 ameliorates lipid peroxidation and neuronal damage in ECM mice. **a** Levels of MDA in the whole brain of control, ECM, and ECM mice treated with Fer-1 were measured with an MDA assay kit. Relative levels are shown as fold-changes in relation to control levels. Each point represents one animal. **b** Expression of NeuN (green) in the cerebrum of control, ECM, and Fer-1-treated ECM mice was detected by immunofluorescence staining and quantified based on fluorescent density. The nucleus was stained with DAPI (blue). Relative fluorescent density is shown as fold-change in relation to levels in control mice. Data are reported as the mean ± standard deviation. Each point represents one region of the immunofluorescence staining image. **p < 0.01 and ***p < 0.001 *vs.* control or ECM. *ECM* experimental cerebral malaria, *Fer-1* ferrostatin-1, *MDA* malondialdehyde, *NeuN* neuronal nuclei

As our results suggested that ferroptosis could contribute for the neuronal damage in ECM, we then explored the protective effect of ferroptosis inhibition. Noteworthily, the MDA content in ECM mice treated with ferroptosis inhibitor Fer-1 almost returned to normal levels (ECM, *p* = 0.001; ECM + Fer-1, *p* = 0.0034) (Fig. 3a); thus, Fer-1 prevented lipid peroxidation in ECM. In addition, immunofluorescence staining revealed that NeuN levels were partially restored in the cerebrum of ECM mice treated with Fer-1 (*p* < 0.0001) (Fig. 3b). These results indicate that neuronal ferroptosis occurs in ECM and its inhibition may protect neurons from ferroptotic damage.

### Activated CD8^+^ T cells may be inducers of neuronal ferroptosis in ECM

**Fig. 4:**
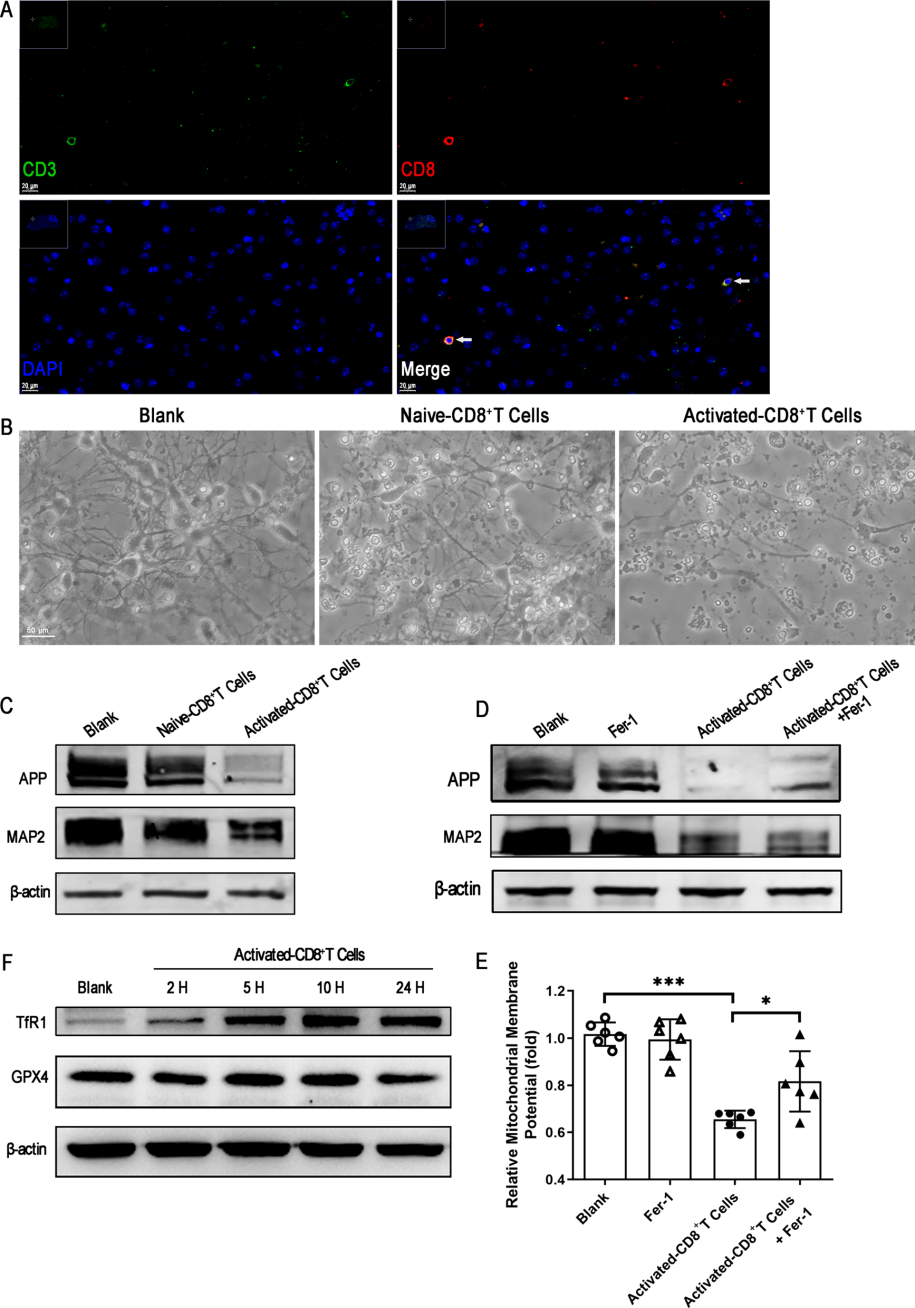
CD8^+^ T cells infiltrate the cerebrum of ECM mice and induce ferroptotic damage to primary neurons. **a** CD8^+^ T cells infiltrating the cerebrum of ECM mice were co-labeled with CD3 (green) and CD8 (red), and detected by immunofluorescence staining. The nucleus was stained with DAPI (blue). **b** Morphological changes in primary neurons were observed by microscopy (200 × magnification). **c** Expression of APP and MAP2 in primary neurons, as measured by western blotting, after co-culture with naïve or activated CD8^+^ T cells. **d** Expression of APP and MAP2 in primary neurons, as measured by western blotting after co-culture with activated CD8^+^ T cells with or without Fer-1. **e** Relative mitochondrial membrane potential (MMP) levels were calculated based on the optical density value and shown as the fold change relative to the blank value. Data are reported as mean ± standard deviation. Each point represents one sample. *p < 0.05 and ***p < 0.001 *vs.* control or activated CD8^+^ T cells. **f** Expression of TfR1 and GPX4 in primary neurons, measured by western blotting after co-culture with activated CD8^+^ T cells for 2–24 h. *APP* amyloid-beta precursor protein, *ECM* experimental cerebral malaria, *GPX4* glutathione peroxidase 4, *MAP2* microtubule-associated protein 2, *TfR1* transferrin receptor 1

Previous single-cell RNA sequencing analysis of ECM mouse brain stem tissues revealed that a large number of activated CD8^+^ T cells infiltrated the brain parenchyma, already reported by our group[24]. In agreement with this data, many CD8^+^ T cells were detected in the cerebrum of ECM mice (Fig. 4a), which suggested that activated CD8^+^ T cells may be a potential inducer of neuronal ferroptosis in ECM. Next, we cultured primary cortical neurons and co-cultured them with activated CD8^+^ T cells from ECM mice to further investigate whether these immune cells could contribute for neuronal ferroptosis. Interestingly, dendrites and cell bodies of neurons were seriously damaged in the presence of activated CD8^+^ T cells, but only slight morphological changes were observed when they were treated with naïve CD8^+^ T cells from control mice as a negative control (Fig. 4b). Furthermore, the expression of the amyloid precursor protein (APP), a transmembrane protein expressed in neurons and shown to promote synapse formation and dendritic sprouting [25], decreased significantly in primary neurons co-cultured with activated CD8^+^ T cells (Fig. 4c). Consistently, the microtubule-associated protein 2 (MAP2), which is expressed in the cell bodies and dendrites of neurons, and has been reported to be reduced in damaged neurons [26, 27], was also shown to be downregulated (Fig. 4c).

Lastly, we tested the protective effect of a ferroptosis inhibitor on neuronal APP expression and MMP, which is also an indicator of ferroptosis, which can be caused by elevated intracellular reactive oxygen species generated from L-OOH. Fer-1 partially rescued APP expression in neurons co-cultured with activated CD8^+^ T cells, but was unable to rescue MAP2 expression (Fig. 4d). As expected, MMP levels in primary neurons were significantly decreased in the presence of activated CD8^+^ T cells (*p* < 0.0001), and pretreatment with Fer-1 partially reversed this event (*p* = 0.0144) (Fig. 4e). These results indicate that activated CD8^+^ T cells induce ferroptosis in neurons and partially contribute to neuronal damage, and Fer-1 could protect neurons from this ferroptotic damage.

Further, TfR1 expression was significantly upregulated in neurons co-cultured with activated CD8^+^ T cells in a time dependent manner (2–24 h). Unexpectedly, no obvious change was detected in GPX4 expression, except for a slight reduction when neurons were co-cultured with activated CD8^+^ T cells for 24 h (Fig. 4f). These results suggest that infiltrating activated CD8^+^ T cells can induce TfR1 upregulation in the ECM mouse brain and consequently promote neuronal ferroptosis. However, the decreased GPX4 expression in neurons of ECM mice, seems not to be induced by infiltrating activated CD8^+^ T cells.

## Discussion

Ferroptosis is a newly discovered form of programmed cell death, which is dependent on oxidative stress and mediated by iron-dependent lipid peroxidation. In some disorders, cells in a high oxidative stress state can produce excessive L-OOH, which promotes susceptibility to ferroptosis [28, 29]. Studies have reported that neuronal damage or death is closely correlated with ferroptosis in many central nervous system diseases, resulting in neurological sequelae [30–34]. Recent studies have revealed that oxidative stress is involved in CM and ECM [35–37], but its potential role and pathogenic mechanisms are not completely understood. Thus, we hypothesized that ferroptosis could contribute to neuronal damage and facilitate the occurrence of neurological sequelae in patients with CM.

Herein, we demonstrated that neurons were damaged in ECM mice, with concomitant increased levels of Fe and L-OOH. Moreover, the susceptibility of ECM neurons to ferroptosis was further confirmed by increased TfR1 and ACSL4 levels, and reduced GPX4 expression, mainly in the cerebrum. TfR1 is the critical receptor responsible for importing Fe into cells, contributing to ferroptosis [38, 39], and is a specific ferroptosis marker [40]. Many studies have revealed that ACSL4 is an important lipoxygenase and a critical determinant of ferroptosis sensitivity [18, 41]. However, TfR1 and ACSL4 have not been investigated in ECM-associated ferroptosis. We demonstrated that ACSL4 expression is increased in all brain regions, and TfR1 expression is increased in the olfactory bulb and cerebrum of ECM mice. From our results, high Fe content in the brain tissue provides a favorable environment for TfR1-induced intracellular Fe accumulation and upregulated ACSL4 can catalyze L-OOH production more vigorously, which together contributes to ferroptosis in the ECM mice.

In addition, significant downregulation of GPX4 expression was observed in the olfactory bulb and cerebrum of the ECM mice. Importantly, reduced GPX4 expression was positively correlated with damaged neurons. GPX4 is a key antioxidant enzyme that neutralizes L-OOH and plays an important role in regulating ferroptosis [42]. GPX4 inhibition by erastin and RSL3 is a general method used to induce ferroptosis [43, 44]. Indeed, deficient GPX4 expression in neurons can cause ferroptotic damage in experimental autoimmune encephalomyelitis models and cause significant neurological sequela [31, 45]. Therefore, we believe that reduced GPX4 expression can be an important ferroptosis regulator and cause neuronal damage in ECM. Noteworthily, the ECM-associated ferroptotic damage to neurons was further confirmed by the administration of the ferroptosis inhibitor Fer-1, which ameliorated lipid peroxidation and neuronal damage in ECM mice.

Unexpectedly, GPX4 expression was found to be upregulated in the brainstem of ECM mice. One possible reason for this is that GPX4 is upregulated by the excessive L-OOH accumulation in the brainstem in ECM under a negative feedback regulatory mechanism. This result is in line with those of a recent study in which ferroptotic cell death occurred even when increased GPX4 expression was detected after intracerebral hemorrhage, possibly due to an inadequate attempt of the cell to protect itself [46]. However, this possibility needs to be further studied.

Lastly, to study the potential inducer of neuronal ferroptosis in ECM mice, we co-cultured primary neurons with activated CD8^+^ T cells, which were found to markedly infiltrate the brain parenchyma of ECM mice. Our data indicated that activated CD8^+^ T cells significantly damaged neurons, promoting serious morphological changes, reduced APP and MAP2 expression, and decreased MMP. Although APP is infamous for its pivotal role in the pathogenesis of Alzheimer’s disease [47], it has been reported to regulate synapse formation and function, dendritic sprouting and neuron migration, and neurons derived from APP^−/−^ mice showed significantly decreased dendritic spines and abnormal morphology [48, 49]. In our study, Fer-1 partially rescued APP expression and partially upregulated MMP in neurons co-cultured with activated CD8^+^ T cells, but had little effect on MAP2 expression. One possible reason is that, there are many ways in which neurons are damaged by activated CD8^+^ T cells, including by the action of secreted interferon-γ and granzyme B [50, 51], which may be responsible for inducing decreased expression of APP and MAP2, whereas ferroptosis partially participate in the process, both in vivo and in vitro. Furthermore, early neuronal damage may induce a reduction of APP and MAP2 levels, and lead to ferroptosis at a later stage.

In addition, TfR1 expression was markedly increased in neurons co-cultured with activated CD8^+^ T cells. We speculate that the increased TfR1 expression in the cerebrum of ECM mice was probably induced by infiltrated activated CD8^+^ T cells and that partially contributed to neuronal ferroptosis. CD8^+^ T cells have been reported to be key factors in the pathogenesis of ECM and other central nervous system diseases [50, 52, 53]. A study reported that activated CD8^+^ T cells were commonly recruited to the brain and subsequently induced neuronal death in ECM [53], but our study was the first to indicate that neuronal ferroptosis, which may be induced by activated CD8^+^ T cells, contributes for ECM pathogenesis.

Unexpectedly, significantly reduced GPX4 expression in ECM neurons was not detected in primary neurons co-cultured with activated CD8^+^ T cells. It was reported that neuronal damage can also be caused by glial cells or other inducers, such as excitotoxicity, endoplasmic reticulum stress, and hemin [46, 54–57]. Therefore, we hypothesize that the reduced GPX4 expression is probably caused by other inducers, and this does not deny the key role of GPX4 deficiency in inducing neuronal ferroptosis in ECM pathogenesis.

In conclusion, our study was the first to investigate the role of ferroptosis in the pathogenesis of ECM, showing that neuronal ferroptosis is mainly resultant of TfR1 and ACSL4 upregulation, GPX4 downregulation, and that activated CD8^+^ T cells may be important inducers of ferroptosis. Ferroptotic damage in neurons can be alleviated by ferroptosis inhibitors both in vivo and in vitro. These findings suggest that ferroptotic damage to neurons can be a factor leading to neurological sequelae in CM survivors, paving the way for the development of adjuvant targeted therapy for CM. Nevertheless, further studies are warranted to better understand its specific underlying mechanisms.

## Data Availability

The dataset generated and analyzed during the current study is available from the corresponding author upon reasonable request.

## Abbreviations

ACSL4: Acyl-CoA synthetase long-chain family member 4
APP: Amyloid precursor protein
CM: Cerebral malaria
ECM: Experimental cerebral malaria
Fer-1: Ferrostatin-1
GPX4: Glutathione peroxidase 4
L-OOH: Lipid hydroperoxides
MAP2: Microtubule-associated protein 2
MDA: Malondialdehyde
NeuN: Neuron nuclei
TfR1: Transferrin receptor 1
